# Yoga for posttraumatic stress disorder – a systematic review and meta-analysis

**DOI:** 10.1186/s12888-018-1650-x

**Published:** 2018-03-22

**Authors:** Holger Cramer, Dennis Anheyer, Felix J. Saha, Gustav Dobos

**Affiliations:** Department of Internal and Integrative Medicine, Kliniken Essen-Mitte, Faculty of Medicine, University of Duisburg-Essen, Essen, Germany

**Keywords:** Posttraumatic stress disorder, PTSD, Yoga, Meta-analysis

## Abstract

**Background:**

Yoga is increasingly used as a therapeutic treatment and seems to improve psychiatric conditions such as anxiety disorders and depression. The aim of this systematic review was to assess the evidence of yoga for reducing symptoms of posttraumatic stress disorder (PTSD).

**Methods:**

The Cochrane Library, Medline/PubMed, PsycINFO, Scopus, and IndMED were searched through July 2017 for randomized controlled trials (RCTs) assessing the effects of yoga on symptoms of PTSD. Mean differences (MD) and standardized mean differences (SMD) with 95% confidence intervals (CI) were computed. The quality of evidence and the strength of recommendation were graded according to the GRADE recommendations.

**Results:**

Seven RCTs (*N* = 284) were included. Meta-analysis revealed low quality evidence for clinically relevant effects of yoga on PTSD symptoms compared to no treatment (SMD = − 1.10, 95% CI [− 1.72, − 0.47], *p* < .001, I^2^ = 72%; MD = − 13.11, 95% CI [− 17.95, − 8.27]); and very low evidence for comparable effects of yoga and attention control interventions (SMD = − 0.31, 95%CI = [− 0.84, 0.22], *p* = .25; I^2^ = 43%). Very low evidence was found for comparable retention of patients in the trial for yoga and no treatment (OR = 0.68, 95%CI [0.06, 7.72]) or attention control interventions (OR = 0.66, 95%CI [0.10, 4.46]). No serious adverse events were reported.

**Limitations:**

Few RCTs with only limited sample size were available.

**Conclusions:**

Only a weak recommendation for yoga as an adjunctive intervention for PTSD can be made. More high quality research is needed to confirm or disconfirm these findings.

**Electronic supplementary material:**

The online version of this article (10.1186/s12888-018-1650-x) contains supplementary material, which is available to authorized users.

## Background

With a lifetime prevalence of up to 6.1% worldwide, posttraumatic stress disorder (PTSD) is a major public health problem [1]. PTSD often results from substantial traumatic experiences and is thus far more common among veterans, survivors of wars or natural disasters, and victims of violence. The syndrome is associated with re-experiencing, avoidance, arousal, cognition, and mood symptoms [2].

A wide range of pharmacological and psychotherapeutic approaches are used in the treatment of PTSD [3]. Recent meta-analyses demonstrated that differences between most pharmacological treatments for PTSD and placebo are small at best [4, 5]. While phenelzine seems to be most effective, the paucity of available studies hindered definite conclusions [5]. The strong placebo effect in pharmacological treatment of PTSD might be a main reason for their perceived efficacy [4]. Psychotherapy, especially trauma-focused psychotherapy, seems to be superior to medication as a first-line treatment [6]. While psychotherapy thus seems to be the most promising first-line treatment for PTSD [3], a recent Cochrane Review rated the quality of evidence to be very low even for trauma-focused psychotherapy; and a considerable number of patients terminated the included psychotherapeutic trials early during the treatment period [7]. The latter might hint to a limited tolerability of these treatment approaches in some patients. In recent years, complementary therapy approaches for individuals with PTSD and other trauma-related disorders have received increasing interest [8]. Specifically mind-body approaches might be able to decrease trauma-related symptoms and emotion dysregulation [9]; and could thus be offered when patients are not able to tolerate psychotherapeutic treatment.

The most commonly used mind-body approach is yoga, a traditional Indian philosophical and spiritual school, as well as a health care practice which originated more than 3000 year ago and combines physical activity, mindfulness, relaxation, and breathing exercises [10]. Beside physical aspects, traditional yoga comprises ethical and spiritual perspectives. Modern yoga forms mostly comprise physical poses, breathing exercises, and meditation. Therefore, yoga is a holistic approach, which is thought to help uniting body, mind and spirit [10]. Thus, yoga is increasingly used to foster physical and mental well-being [10]. The efficacy of yoga in improving comorbid mental symptoms could be shown for different health conditions such as pain [11] and cancer [12]. There also is emerging evidence of efficacy of yoga as an ancillary treatment for psychiatric conditions such as depression [13–15], anxiety disorders [16, 17], and perhaps psychosis [18–20]. Yoga has become one of the most commonly used complementary therapy approaches in Northern America [21]. With respect to potential mechanisms of action, physical activity is a major element in yoga and this physical activity alone already seems to improve PTSD symptoms [22]. It has however been argued that by reducing stress-induced allostatic load and increasing parasympathic activity, yoga can directly reduce amygdala hyperactivation and elevated cortisol levels in patients with PTSD and thereby reduce symptoms [23]. This shift in autonomic balance to the parasympathic branch of the autonomic nervous system seems to be mainly triggered by breathing, relaxation, and meditation which are specific to yoga interventions [24]. Beyond biological mechanisms, yoga might potentially impact PTSD via different psychological pathways. Two major mechanisms of the paradox that patients feel anxious about the future although the traumatic event lies in the past were identified: negative appraisals of the trauma or its sequelae and the nature of the trauma memory itself [25]. Negative appraisals include overgeneralization, negative appraisal of own actions, negative reactions by other people and life prospects. These different types of appraisals can trigger the different emotional experiences by patients with PTSD [25]. Yoga often involves aspects of mindfulness, ie, a non-judgmental mindful attention to and acknowledgment of even unpleasant emotions or memories [26]. This is thought to increase emotion regulation rather than avoidance. The mindful awareness of the transitory nature of one’s momentary physical, sensory, and emotional experience during yoga practice is thought to lead to a change in self-appraisal [27] and to also induce positive effects on PTSD symptoms [28]. Furthermore, even unspecific relaxation has been shown to contribute to an amelioration of PTSD symptoms although to a much smaller extent than more specific psychotherapeutic approaches [29]. Physical activity in yoga is mainly associated with an increased attentional focus to bodily perceptions and sensations. This can increase body awareness and potentially accurate identification of the triggered emotional response [30]. Beyond that, the social support in yoga classes might positively influence negative cognitive appraisals [31].

This systematic review aimed to investigate and meta-analyze the evidence of yoga for symptoms of PTSD.

## Method

In conducting and reporting of this review, the Preferred Reporting Items for Systematic Reviews and Meta-Analyses (PRISMA) [32] statement and the Cochrane Collaboration’s recommendations [33] were applied. The protocol was not published a priori.

### Eligibility criteria

Types of studies: Randomized controlled trials (RCTs) were eligible. Studies were eligible regardless of the language they were published in, their country of origin or sample size.

Types of participants: Studies on adults with PTSD diagnosed by an established clinician-administered instrument and/or using a validated self-report instrument were eligible. Studies involving participants with comorbid physical or mental disorders were eligible for inclusion. Studies also including participants without PTSD were excluded unless outcomes for patients with PTSD were reported separately.

Types of interventions: RCTs on yoga were eligible. Studies were eligible regardless of the specific yoga style, the frequency or length of the intervention program or the duration of yoga sessions.

Types of control interventions: RCTs comparing yoga to no treatment, attention control (‘placebo’ interventions controlling for therapists’ attention without a presumed specific therapeutic component) or other comparators were eligible. Meta-analyses were conducted separately for the different control conditions.

Studies allowing participants to use individual co-interventions (such as pharmacotherapy) were eligible as long as all participants in all group received the same co-interventions.

Types of outcome measures: Studies assessing symptoms of PTSD by at least one validated instrument were eligible. Where multiple instruments for PTSD symptoms were used in a single study, primacy was given to the instrument used in most of the other studies to increase comparability and homogeneity. If instruments were only used in a single study, primacy was given to clinician-assessed instruments over self-report measures.

Retention of patients in the trial and safety were assessed as secondary outcomes. Retention in the trial was defined as the number of patients who did not terminate the trial early during the treatment period; safety was defined as the number of patients who experienced adverse events.

### Search methods

The electronic databases the Cochrane Library, Medline/PubMed, PsycINFO, Scopus, and IndMED were searched from their inception until July 31, 2017. The complete search strategy for all databases is shown in Additional file 1. Our own extensive database [34] was searched manually, as were the reference lists of articles identified during literature search and the tables of contents of the the Journal of Yoga & Physical Therapy, International Journal of Yoga Therapy, and the International Scientific Yoga Journal SENSE. The PILOTS database was additionally searched. Two reviewers independently assessed abstracts and potentially eligible articles identified during literature search. Discrepancies were resolved by discussion; if necessary a third reviewer was involved.

### Data extraction and management

Data on participants, methods, interventions, control interventions, outcomes, and results were extracted by two review authors independently. An a priori developed data extraction form was used. Discrepancies were resolved by discussion; if necessary a third reviewer was involved.

The same two reviewers independently assessed risk of bias using the Cochrane tool [33]. Using this tool, risk of bias is assessed on seven domains including random sequence generation, allocation concealment, blinding of participants and personnel, blinding of outcome assessment, incomplete outcome data, selective reporting, and other bias. All domains were scored as low risk of bias, unclear risk of bias, or high risk of bias. No summary score is computed but all domains are assessed individually. Discrepancies were resolved by discussion; if necessary a third reviewer was involved.

### Data analysis

For PTSD symptoms, meta-analyses were computed using standardized mean differences (SMD) and their respective 95% confidence intervals (CI) by a random-effects inverse variance model [33]. Using Hedges’ correction for studies with small sample size, SMDs were computed as the between-group difference in means which is divided by the pooled standard deviation. When the original studies reported no standard deviations, they were calculated from standard errors, CIs or t-values. In line with the approach used by the Cochrane Collaboration, a negative SMD was regarded to indicate fewer PTSD symptoms in the yoga group compared to the control group [33]. Cohen’s categories were used to estimate the size of the overall effect: SMDs ranging from 0.2 to 0.5 were defined as small effects; SMDs ranging from 0.5 to 0.8 were defined as medium size effects and SMDs beyond 0.8 were defined as large effect sizes [33, 35]. Where the same assessment instrument was used in multiple studies, mean differences and their 95% CI were further calculated as the absolute difference between mean values in the two groups [33]. For retention of patients in the trial, odds ratios (OR) with 95% confidence intervals (CIs) were calculated [33]. We originally planned to also calculate OR for safety; due to the paucity of studies reporting adequate safety-related data, this could not be done.

All analyses were computed using Review Manager 5 software (Version 5.3, The Nordic Cochrane Centre, Copenhagen).

As a measure of heterogeneity between studies, the I^2^ statistics and Chi^2^ test were used. Given the low power of this test, when only few studies or studies with low sample sizes are included in a meta-analysis, a *p*-value ≤0.10 was regarded as indicating significant heterogeneity [33].

Taking the quality of the respective studies as well as the confidence in the results into account, we graded the quality of evidence and the strength of recommendation. The recommendations of the Grading of Recommendations Assessment, Development and Evaluation (GRADE) working group were used. The quality of evidence was graded as high quality, moderate quality, low quality or very low quality based on the limitations of the respective studies, the inconsistency between the results of the respective, the indirectness of the evidence, the imprecision of the findings, the risk of publication bias, as well as the risk of other bias [36]. A body of evidence obtained from RCTs is initially graded as high quality, this rating can be downgraded due to risk of methodological bias, heterogeneity, differences in study characteristics between studies, wide confidence intervals, and risk of publication bias. Importantly, GRADE does not evaluate the quality of single studies but the quality of the complete body of evidence; e.g. the evidence for effects of yoga compared to a specific comparator on PTSD symptoms is rated [36]. A high quality of evidence indicates a high confidence that the effect found in the meta-analysis is a relatively good estimator for the true. A moderate quality indicates that the effect found in the meta-analysis is likely to be a good estimator for the true effect, however the true effect might also differ substantially from it. A low quality indicates that the effect found in the meta-analysis may differ substantially from the true effect. A very low quality indicates that the effect found in the meta-analysis will likely substantially differ from the true effect [37]. Finally, the strength of recommendation for or against yoga as an intervention for PTSD was judged as either strong or weak according to the GRADE recommendations. This judgement was based on the direction of the evidence (in favor of yoga or in favor of the control interventions), the quality of the evidence (see above) and the risk of adverse events of the interventions [36].

We conducted subgroup analyses to assess effects for different types of interventions (yoga using physical poses or yoga using no physical poses).

### Risk of bias across studies

We had planned to investigate risk of publication bias by visual judgement of funnel plot asymmetry using Review Manager Software [33, 38]. As this approach requires at least 10 studies to be include in a respective meta-analysis, and all analyses included less than 10 studies, this could not be done.

## Results

### Literature search

**Fig. 1:**
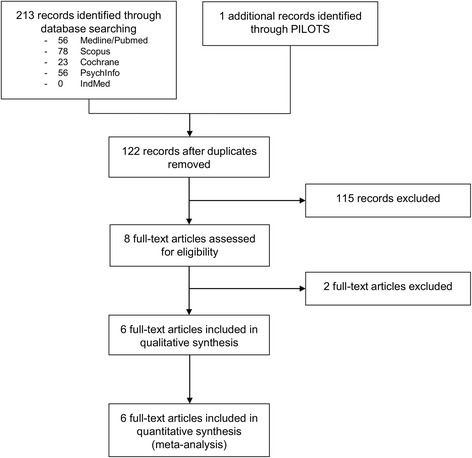
Study flow diagram

Out of 282 records identified (143 after duplicates removed), 9 full-texts were assessed [39–47]. Two full-texts were excluded because they were additional publications on already included RCTs reporting additional outcomes [46] or follow-up assessments [47]. Seven RCTs on 284 patients were included in the qualitative and quantitative review [40–45] (Fig. 1).

### Study characteristics

Five of the included RCTs originated from North America [40, 41, 43–45], one from South America [42], and one from Australia [39]. The included patients in three RCTs were diagnosed by the self-report Posttraumatic Stress Disorder Checklist [40, 42, 44] while four RCTs used clinician-assessed instruments [39, 41, 43, 45]. The majority of included patients were male (median = 73%), mean age ranged from 28.7 to 58.0 years (median = 43.6 years) (Table 1). Four RCTs included only military personnel and/or veterans [39, 42–44]. RCTs were roughly comparable regarding duration (median = 9.5 weeks) and intensity of the intervention (median = 1.75 sessions per week). Only one RCT used a staged intervention starting with daily, then weekly, then monthly sessions [39]. A variety of yoga styles was used, only one of them (applied in two RCTs) was specifically designed for patients with an experience of trauma [41, 45]. All but one yoga intervention [44] included physical postures. Four RCTs used waitlist control groups without any specific treatment; two RCTs used attention control interventions which controlled for therapists’ time and attention but without a specific therapeutic component, i.e., an unspecific women’s health education program and a weekly assessment group session without a therapeutic component [41, 45] (Table 1). A further study used a so-called assessment-only control, but while it used a group-assessment session, this session only took place once before and after the intervention while the intervention group met twice weekly over 10 weeks, so we classified this control condition as no treatment [43].

**Table 1:** Characteristics of included studies

Author, year	Participants and setting	*N*	Diagnostic instrument	Mean age	% female	Treatment group	Control group	Outcome measures
Carter et al., 2013 [36]	Australian veterans recruited from private psychiatry clinic and through advertisements	31	CAPS	58.5 years	0%	Sudarshan Kriya Yoga: 22 h within 5 days, then once weekly for one month, then once monthly for 5 months, 120 min each	Waitlist: 6 weeks	CAPS, PCL-M
Jindani et al., 2015 [40]	Canadan community-dwelling adults recruited through advertisements	80	PCL-17	41.00 years	89%	Kundalini Yoga: 8 weeks, once weekly, 90 min each	Waitlist: 8 weeks	PCL-17
Mitchell et al., 2014 [41]	Veterans and civilians recruited from a US Veteran Affairs medical center	38	PSS-I	44.37 years	100%	Kripalu-based Yoga: 6 or 12 weeks, once or twice weekly, 75 min	Assessment control: 12 weeks, once weekly, duration not reported	PCL-C, TLEQ
Quinones et al., 2015 [42]	Colombian reintegrating PTSD patients recruited through the Agencia Colombiana para la Reintegración	100	PCL-C	Not reported	27%	Satvananda Yoga: 16 weeks, twice weekly, 60 min	Waitlist: 16 weeks	PCL-C
Reinhardt et al., 2017 [43]	US active duty military personnel and veterans recruited by flyers and advertisement	51	SCID-CT	46.74 years	8%	Kripalu Yoga: 10 weeks, twice weekly, 90 min	Assessment control: 10 weeks, three times in total, duration not reported	PCL-M, PCL-C, CAPS, IES
Seppälä et al., 2014 [44]	US veterans recruited through flyers and veteran and military organizations	21	PCL-M	28.65 years	0%	Sudarshan Kriya Yoga: 7 days, once daily, 180 min	Waitlist: 7 days	PCL-M
Van der Kolk et al., 2014 [45]	US women recruited through advertisements and mental health professionals	64	CAPS	42.9 years	100%	Trauma-informed Yoga: 10 weeks, once weekly, 60 min	Women’s health education: 10 weeks, once weekly, 60 min	CAPS, DES, DTS

*CAPS* Clinician-Administered Posttraumatic Stress Disorder Scale, *DES* Dissociative Experience Scale, *DTS* Davidson Trauma Scale, *IES* Impact of Events Scale, *PCL-17* Posttraumatic Stress Disorder Checklist, *PCL-C* Posttraumatic Stress Disorder Checklist-Civilian, *PCL-M* Posttraumatic Stress Disorder Checklist-Military, *PSS-I* PTSD Symptom Scale-Interview, *SCID-CT* Structured Clinical Interview for DSM-IV-TR Axis I Disorders, clinical trials version, *TLEQ* Trauma Life Events Questionnaire

**Fig. 2:**
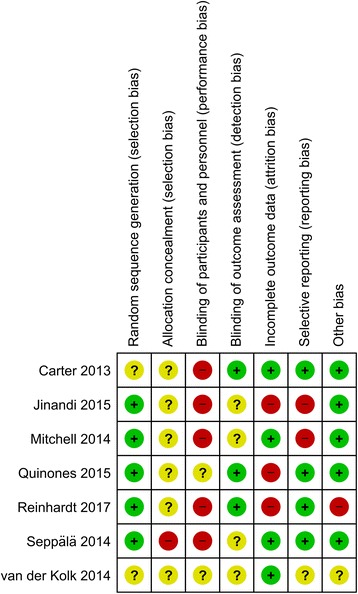
Risk of bias assessment for each included study. +, low risk of bias; −, high risk of bias; ?, unclear risk of bias

Risk of bias was mixed (Fig. 2). While five RCTs used adequate methods of random sequence generation [40, 41, 43–45], none adequately concealed allocation and only three RCTs blinded outcome assessors [39, 42, 43]. Five RCTs explicitly reported to not have blinded patients to treatment allocation [39–41, 43, 44]; the remaining RCTs did not report on blinding of participants and personnel. Given the difficulties of blinding behavioral interventions, participants and personnel were most likely not blinded to the allocated intervention in these trials. Attrition was high and not well balanced between groups in three RCTs and no adequate intention-to-treat analysis was used [40, 42, 43]. Finally, one study was rated as having high risk of other bias because it offered a substantial financial compensation to control participants but not to participants in the yoga group [42]. This methodologic approach most likely resulted in the strong imbalance in dropouts between groups.

### Outcomes

**Fig. 3:**
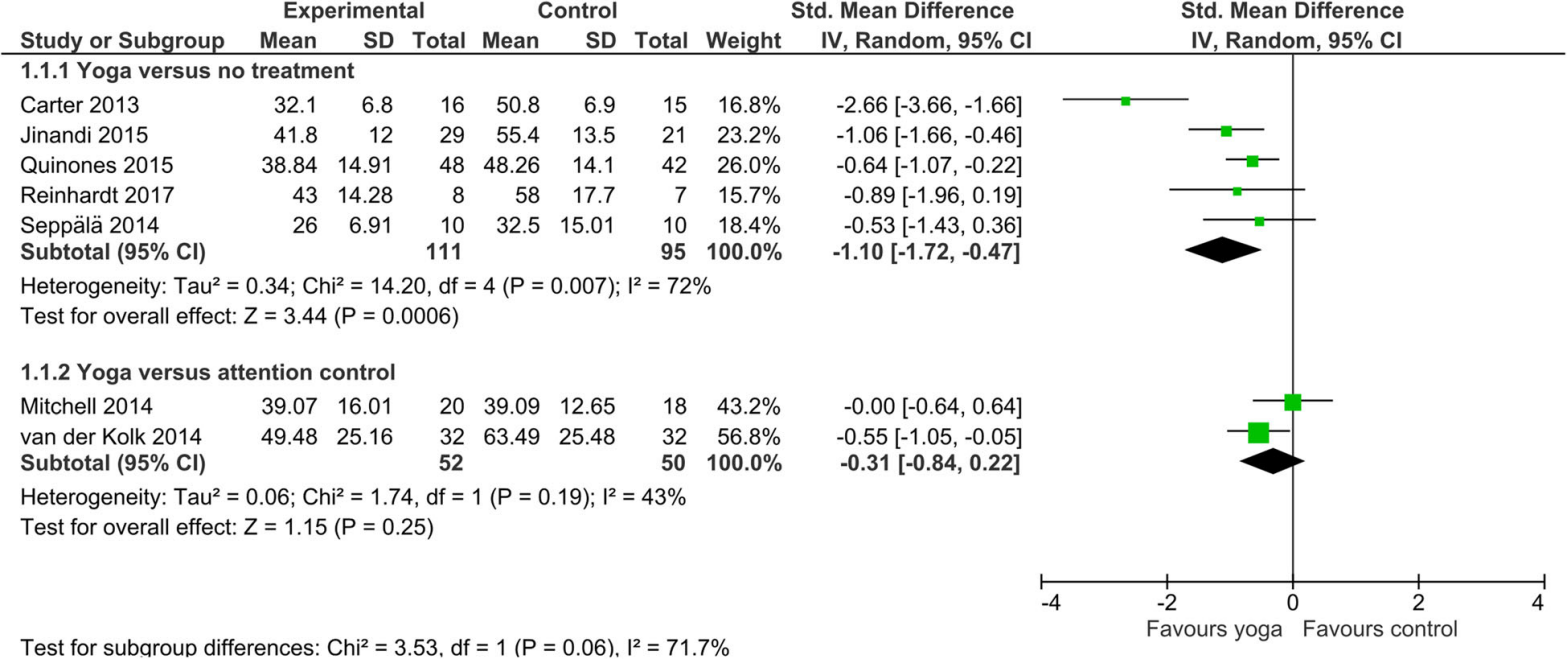
Effects of yoga versus no treatment or attention control interventions on PTSD symptoms. CI, confidence interval; IV, inverse variance; SD; standard deviation

Meta-analysis revealed evidence for statistically significant effects of yoga on PTSD symptoms compared to no treatment (SMD = − 1.10, 95% CI [− 1.72, − 0.47], *p* < .001; Fig. 3), with significant heterogeneity (I^2^ = 72%, *p* = .007). All studies used the PTSD checklist. The mean difference (MD = − 13.11, 95% CI [− 17.95, − 827]) was above the defined threshold of 10 points indicating clinical relevance [48], while the confidence interval also included values below the threshold for clinical relevance. The quality of evidence was downgraded to low due to high likelihood of bias, imprecision of results and the unclear risk of publication bias. The results did not change substantially when only those RCTs were considered whose yoga intervention included physical postures (SMD = − 1.24, 95% CI [− 1.99, − 0.48], *p* < .001; heterogeneity: I^2^ = 78%, *p* = .004). The effects were however no longer present when the single RCT was considered that did not include physical postures (SMD = − 0.53, 95% CI [− 1.43, 0.36], *p* = .24).

Meta-analysis found no group differences of yoga compared to attention control interventions (SMD = − 0.31, 95%CI = [− 0.84, 0.22], *p* = .25; Fig. 3), without significant heterogeneity (I^2^ = 43%, *p* = .19). The quality of evidence for this lack of effects was downgraded to very low because of high likelihood of bias, unexplained heterogeneity, imprecision of results, and unclear risk of publication bias. Effects did not differ between the comparisons of yoga versus no treatment and yoga versus attention control (Chi^2^ = 3.53, *P* = 0.06, I^2^ = 71.1%).

**Fig. 4:**
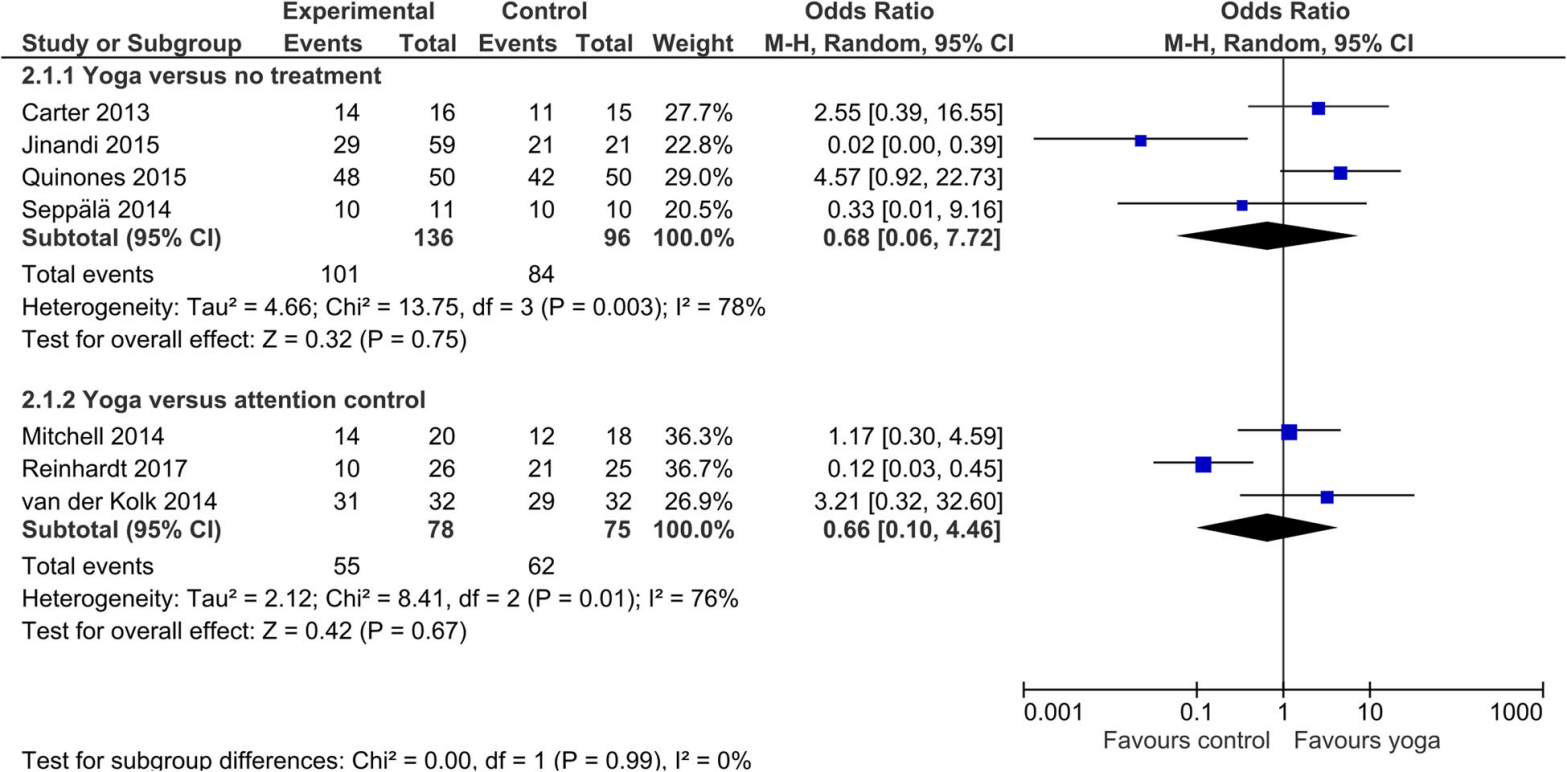
Retention of patients in the trial for yoga versus no treatment or attention control interventions. CI, confidence interval; IV, inverse variance; SD; standard deviation

Retention of patients in the trial was comparable between yoga and no treatment (OR = 0.68, 95%CI = [0.06, 7.72], *p* = 0.75) and between yoga and attention control (OR = 0.66, 95%CI = [0.10, 4.46], *p* = 0.67) (Fig. 4). The quality of evidence for this lack of differences was downgraded to very low because of high likelihood of bias, unexplained heterogeneity, imprecision of results, and unclear publication bias. Retention did not differ between the comparisons of yoga versus no treatment and yoga versus attention control (Chi^2^ = .00, *p* = .99, I^2^ = 0%).

The exported data and analyses of the meta-analyses are provided in Additional file 2.

Only three RCTs reported any safety-related data; and reported that no adverse reactions [41], no serious adverse events occurred [42], or reported two exacerbations of preexisting breathing problems in the yoga group while not reporting on adverse events in the control group [39].

## Discussion

This meta-analysis found low quality evidence for statistically significant and potentially clinically relevant effects of yoga on symptoms of PTSD. While yoga was not superior to attention control interventions, the true effect is likely to be substantially different from the estimated effect [37]. Retention of patients in the trial differed widely between studies, but overall, dropouts were comparable between yoga and the control interventions. The evidence for this non-significant group difference was very low. It should however be noted that low and very low quality of evidence is a common finding when using GRADE methodology in meta-analyses of non-pharmacological interventions for PTSD [e.g. 7]. While safety was insufficiently reported, yoga did not seem to be associated with serious adverse events. The RCTs included veterans and civilians, a wide age range and patients of both genders. The applicability of the findings however is limited by the fact that all but one study were conducted in North America. Regarding intervention types, the effects seem to be only applicable to yoga interventions which include physical postures but not to mainly breathing-based yoga intervention without physical postures.

The findings of this meta-analysis are partly in line with that of a recent meta-analysis demonstrating a small to medium-size effect of yoga and meditation on PTSD symptoms [49]. While the analysis found no significant effects when only so called yoga based approaches were considered, it included one non-yoga study in their analysis [50]. It moreover pooled a variety of control conditions in a single analysis, rendering interpretation of their findings difficult and hindering direct comparison to our analysis. The findings of our meta-analysis are further in line with that of a recent meta-analysis on meditation that found evidence for positive effects across different meditation types including yoga [51]. In line with our findings, a recent qualitative review on emerging PTSD interventions found moderate quality evidence for positive effects of yoga on PTSD symptoms [52]. Further narrative non-systematic reviews found comparable results [53, 54].

A limitation of this review is the diagnosis method used in the included studies. Only four trials used an established clinician-administered instrument [39, 41, 43, 45] while the others relied on potentially less precise self-report measures. Future studies should ensure adequate diagnostic criteria. The small number of studies included in this systematic review, the mixed risk of bias and the inadequate reporting of safety-related data further limit the validity of the findings. Future studies of yoga for PTSD should ensure rigorous trial design and reporting, mainly adequate allocation concealment and blinding of at least outcome assessors [55]. At the moment, yoga can only be considered as an adjunct to other established treatments such as exposure therapy. If yoga is thought to be considered as a stand-alone intervention for PTSD, it should be empirically tested to an equal degree as established therapies [56]. Future studies should then further compare yoga to established therapies for PTSD to assess their comparative effectiveness. Standardization is an important question in yoga intervention and as shown in this meta-analysis, not all yoga styles can be considered equally effective for PTSD. In line with this, the active components of yoga interventions remain unclear; dismantling studies would be needed to assess the individual effects of yoga postures, breathing exercises and meditation/relaxation. An essential question for future studies on yoga for PTSD is the development of adequate control conditions. As shown, the two included studies found no difference between yoga and attention control. While this might also be due to a lack of power, more studies using adequately designed attention-control interventions are needed to conclusively address this issue. Comparing yoga to relaxation, exercise or support groups might further test the particular contributions of these intervention components to the overall effect of yoga [56].

## Conclusions

There is low quality evidence that yoga interventions including physical postures could be an effective, acceptable and safe intervention for PTSD. According to the GRADE guidelines, only a weak recommendation for the use of yoga as an adjunctive intervention for PTSD can be made because the true effect may be substantially different from the effect estimated in this analysis [37]. Therefore, more high quality studies are needed to confirm or disconfirm these findings.

## Additional files

Additional file 1: Search strategy. (DOCX 15 kb)

Additional file 2: Exported data and analyses of the meta-analyses. (CSV 2 kb)

## Abbreviations

CAPS: Clinician-administered posttraumatic stress disorder scale
CI: Confidence interval
DES: Dissociative experience scale
DTS: Davidson trauma scale; IES, impact of events scale
GRADE: Grading of recommendations assessment, development and evaluation
MD: Mean differences
PCL-17: Posttraumatic stress disorder checklist
PCL-C: Posttraumatic stress disorder checklist-civilian
PCL-M: Posttraumatic stress disorder checklist-military
PRISMA: Preferred reporting items for systematic reviews and meta-analyses
PSS-I: PTSD Symptom Scale-Interview
PTSD: Posttraumatic stress disorder
RCT: Randomized controlled trial
SCID-CT: Structured Clinical Interview for DSM-IV-TR Axis I Disorders, clinical trials version
SMD: Standardized mean differences
TLEQ: Trauma life events questionnaire
