# Supplementary material for: Yoga for posttraumatic stress disorder – a systematic review and meta-analysis
Source: BMC Psychiatry. 2018 Mar 22;18:72. doi: 10.1186/s12888-018-1650-x (PMC5863799; doi:10.1186/s12888-018-1650-x)
Supplement: Supplementary file 1 — Search strategy. (DOCX 15 kb) [file 12888_2018_1650_MOESM1_ESM.docx]

Additional file 1: Search strategy.

| PubMed | |
| --- | --- |
| #1 | yoga[MeSH Terms] OR yoga[Title/Abstract] OR yogic[Title/Abstract] |
| #2 | stress disorders, post-traumatic[MeSH Terms] OR post-traumatic[Title/Abstract] OR PTSD[Title/Abstract] |
| #3 | #1 AND #2 |
| Scopus | |
| #1 | (TITLE-ABS-KEY(yoga) OR TITLE-ABS-KEY(yogic)) |
| #2 | (TITLE-ABS-KEY(post-traumatic) OR TITLE-ABS-KEY(PTSD)) |
| #3 | #1 AND #2 |
| Cochrane Library | |
| #1 | Yoga:ti,ab,kw or Yogic:ti,ab,kw (Word variations have been searched) |
| #2 | MeSH descriptor: [Yoga] explode all trees |
| #3 | #1 OR #2 |
| #4 | Post-traumatic:ti,ab,kw or PTSD:ti,ab,kw (Word variations have been searched) |
| #5 | MeSH descriptor: [stress disorders, post-traumatic] explode all trees |
| #6 | #4 OR #5 |
| #4 | #3 AND #6 |
| IndMed | |
| #1 | (yoga OR yogic OR) AND (post-traumatic OR PTSD) |
